# Revelation of graphene-Au for direct write deposition and characterization

**DOI:** 10.1186/1556-276X-6-424

**Published:** 2011-06-15

**Authors:** Shweta Bhandari, Melepurath Deepa, Amish G Joshi, Aditya P Saxena, Avanish K Srivastava

**Affiliations:** 1 National Physical Laboratory, Council of Scientific and Industrial Research, Dr. K.S. Krishnan Road, New Delhi, 110 012, India; 2 Department of Chemistry, Indian Institute of Technology Hyderabad, Hyderabad, 502205, India

## Abstract

Graphene nanosheets were prepared using a modified Hummer's method, and Au-graphene nanocomposites were fabricated by *in situ *reduction of a gold salt. The as-produced graphene was characterized by X-ray photoelectron spectroscopy, ultraviolet-visible spectroscopy, scanning electron microscopy, and high-resolution transmission electron microscopy (HR-TEM). In particular, the HR-TEM demonstrated the layered crystallites of graphene with fringe spacing of about 0.32 nm in individual sheets and the ultrafine facetted structure of about 20 to 50 nm of Au particles in graphene composite. Scanning helium ion microscopy (HIM) technique was employed to demonstrate direct write deposition on graphene by lettering with gaps down to 7 nm within the chamber of the microscope. Bare graphene and graphene-gold nanocomposites were further characterized in terms of their composition and optical and electrical properties.

## Introduction

Graphene, structurally known as a monatomic layer of allotropic-carbon atoms in a hexagonal honeycomb two-dimensional lattice system, has always been a potential candidate for various applications due to its remarkable structural, physical, and electronic properties [1-9]. The zero density of state at the Fermi level without an energy gap offered by graphene, and a linear, rather than parabolic, energy dispersion around the Fermi level has been well understood in the past. The material has also been investigated in a combination with other carbon structures to yield composites with superior properties [10,11].

The composites of metal nanoparticles on graphene sheets also provide a new way to develop catalytic, magnetic, and optoelectronic materials. Moreover, adhesion of such metal nanoparticles to the graphene prevents their aggregation in dry state [12]. Recently, Kamat et al. [13] have used solution-based approach of chemical reduction of AuCl_4_^- ^ions in graphene suspensions to fabricate gold (Au) nanoparticles-graphene hybrid assemblies. In yet another report, Goncalves et al. [12] demonstrated how presence of oxygen functionalities at the graphene surface provides reactive sites for the nucleation and growth of Au nanoparticles (AuNPs**). **These graphene/Au nanocomposites act as potential substrates for surface-enhanced Raman scattering. Min et al. [14] have also used a surface-chemistry-based approach for investigating the influence of surface functionalization on the growth of Au nanostructures on graphene thin films by utilizing various pyrene derivatives containing different functional groups.

But in comparison to these reports, the work presented here highlights a simpler route to obtain stable Au nanoparticles-graphene nanocomposites. It also demonstrates the capability of direct labeling on nanocomposite by use of scanning helium ion microscopy (HIM).

The demonstration of imaging by helium (He) ions is relatively a new technique to characterize the surfaces at sub-nanoscale with extraordinary additional advantages of *in situ *ion lithography, nano-patterning, device prototyping, fabrication of quantum dots, beam-induced chemistry, and milling at nanoscale [15,16]. Such a diverse usage is possible due to the light mass of the He ion and high speed, which results in smaller interaction volume with the surface layers and therefore in better resolution and potential milling feature size. From the perspective of sputtering and patterning, the result is a reduced proximity effect in the surface layer. The light ion mass results in low energy transfer and hence a relatively lower sputtering yield compared to gallium. Exploiting the method of nano-patterning of graphene with helium ions leads considerable promise for a number of applications in nanoscale electronics, optoelectronics, and mechanics. It has been emphasized [17-22] that in an application like high-speed field-effect transistors, there is a strong need for graphene to be patterned at the nanoscale. Patterned graphene can form complex extended geomenies and can be readily contacted electrically, yielding a well-controlled connection between microscale and nanoscale systems and devices.

## Experimental section

Hydrogen tetrachloroaurate (HAuCl_4_) was purchased from Aldrich (St. Louis, MO, USA). Sodium borohydride (NaBH_4_) was acquired from Merck (Darmstadt, Germany). Inorganic transparent electrodes of SnO_2_:F-coated glass (Pilkington, sheet resistance of 14 Ω/sq) were cleaned in a soap solution, 30% HCl solution, double-distilled water, acetone, and trichloroethylene (in that order) prior to use. Deionized water (resistivity ≈ 18.2 MΩ cm) obtained through Milli-Q system, nitric acid (HNO_3_) (Merck), sulfuric acid (H_2_SO_4_) (Merck), and toluene (Spectrochem, Hyderabad, India)were used as solvents.

## Preparation of acid-functionalized graphene

For acid functionalization of graphene, a solution with H_2_SO_4_:HNO_3 _in a 3:1 volume ratio (12 ml H_2_SO_4 _and 4 ml HNO_3_) and 2 g graphite powder was made in a flask and refluxed at 40°C for 16 h. The resulting solution was washed with deionized water till the pH was reduced to 5 or 6. As a result, a black colored solution of acid-functionalized graphene was obtained.

## Preparation of Au-graphene nanostructures

To fabricate Au-graphene nanostructures, Au nanoparticles were synthesized *in situ *in graphene suspension by the reduction of gold(III) complex by NaBH_4_. A concentrated aqueous solution of 0.4 M NaBH_4 _was first mixed with acid-functionalized graphene suspension in toluene. With continuous stirring, 30 mM of HAuCl_4 _was then introduced into this suspension. After continuously stirred for 1 h, the resulting Au-graphene composites were collected by centrifugation and washed with water for three times.

## Characterization techniques

Fourier transform infrared spectra for the films were recorded in reflection mode with a Perkin Elmer GX2000 OPTICA spectrophotometer at 28°C, RH ≈ 50% to 53%. *I*-*V *measurements of films were carried out on Keithley 238 high-current electrometer characterization system. Absorbance (A) spectra were recorded in the 200- to 800-nm wavelength range in a Perkin Elmer Lambda 25 spectrophotometer (Perkin Elmer, Ferdinand-Porsche-ring, Rodgau, Germany). X-ray photoelectron spectroscopy (XPS) spectra were recorded for the as-synthesized graphene samples using a Perkin Elmer 1257 model PHI, Maple Grove, Minnesota, 55311 U.S.A operating at a base pressure of 3.8 × 10^-8 ^Torr at 300 K with a non-monochromatized AlK_α _line at 1,486.6 eV, an analyzer pass energy of 60 eV kept for core level spectra and a hemispherical sector analyzer capable of 25-meV resolution. The overall instrumental resolution was about 0.3 eV. The core level spectra were deconvoluted using a non-linear iterative least squares Gaussian fitting procedure. For all fitting doublets, the FWHMs were fixed accordingly.

Surface morphology of the graphene sheets was studied employing a variable pressure scanning electron microscopy (SEM), model: Zeiss EVO MA10 Carl Zeiss SMT AG, Germany. Nanostructural imaging at high magnifications was carried out using HR-TEM model: FEI-Tecnai G^2 ^F 30 STWIN FEI, Achtseweg Noord 5 5651 GG Eindhoven, Netherlands (operated at the electron accelerating voltage of 300 kV). HR-TEM specimens were prepared by dispersing the graphene films on copper grid of 3.05 mm in diameter having a 200-mesh pore size. Further, the surface topography of graphene and graphene-Au composite films was analyzed by HIM (model: Zeiss ORION Carl Zeiss, NTS Corporation Way, Peabody MA 01960, U.S.A.). The He ion capability of the microscope was used to perform the experiments of nanoscale patterning on the surfaces of graphene.

## Results and discussion

### UV-Vis spectral response

**Figure 1:**
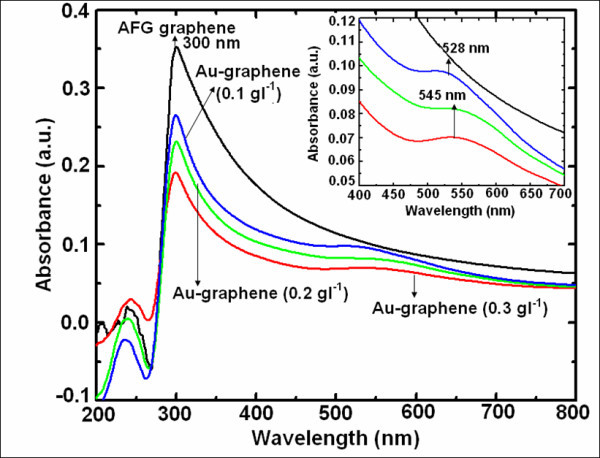
**UV-Vis absorption spectra of pure acid-functionalized graphene and Au-graphene nanocomposites**. In toluene containing different concentrations of functionalized graphene. Inset shows the magnified view of surface plasmon absorption peaks.

The successful synthesis of graphene and Au nanoparticles decorated graphene was confirmed by ultraviolet-visible (UV-Vis) spectroscopy (Figure 1). The UV-Vis spectrum of graphene in toluene shows two absorption peaks, one at 240 nm corresponding to π-π* transitions of aromatic C-C bonds and the other at 300 nm which is attributable to *n*→π* transitions of C=O bonds [23]. When Au nanoparticles were decorated onto the graphene, a broad peak in the visible range was observed corresponding to the surface plasmon absorption of Au nanoparticles. In order to study the effect of graphene concentration in the synthesis of Au nanoparticles, we have also synthesized and recorded UV-Vis spectra at three different concentrations of functionalized graphene in the bath. As concentration of graphene was increased, the peak shows a red shift from 528 nm at 0.1 g l^-1 ^to 545 nm at 0.2 and 0.4 g l^-1^. The quenching in the peak intensity was also observed which is clearly visible in the inset of Figure 1. This is probably attributable to the increase in Au nanoparticle size that further controls the surface plasmon absorption, with increase in the concentration of functionalized graphene [24]. Also, charge transfer from Au nanoparticles to graphene resulted in a decrease in electron density which eventually contributes to the red shift of the surface plasmon absorption [23]. It is highly probable that this charge transfer is playing role in the stability of this nanocomposite.

### X-ray photoelectron spectroscopy

**Figure 2:**
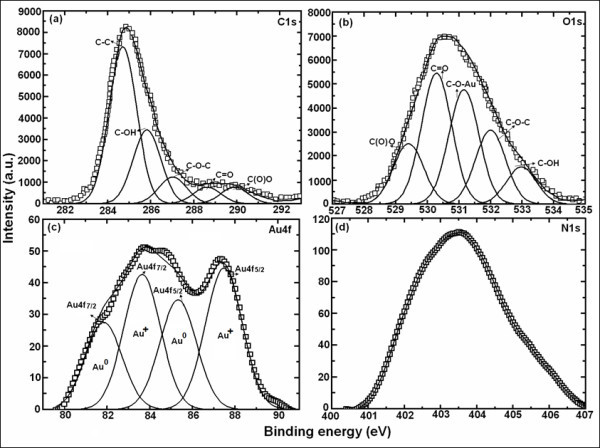
**Core level spectra of Au-graphene nanocomposite**. With solid lines signifying the deconvoluted contributions of (**a**) C1s, (**b**) O1s, (**c**) Au4f and (**d**) N1s.

The formation of stabilized Au-graphene nanocomposite was further confirmed by the XPS spectra as shown in Figure 2. Various compendia of peak attributions of C1s and O1s are listed in Table 1. C1s complex envelope is constituted of five contributions confirming the acid functionalizing of the graphene. Peak at 531.1 eV in O1s spectra owing to C-O-Au bond confirms the stabilized Au-graphene nanocomposite. The N1s peak at 403.5 eV shows clearly the functionalization of graphene by acid treatment. The signature of Au doublet was found with two distinct state of Au(4f_5/2_) and Au(4f_7/2_) [25] due to the spit-orbit splitting. The binding energy values are somewhat lower. Similar trend was observed by Li et al. [23] for Ag/graphene nanocomposites where the effect was attributed to electron transfer from Ag to graphene due to smaller wave function of Ag than graphene [26,27]. Interaction between Au and C=O of graphene also contributes to the electron transfer [28], and the result corroborates with that of UV results. The binding energy difference between the two states found 3.7 eV, which confirms the Au in charged Au^+ ^state. Deconvolution was performed on C(1s), O(1s) and Au(4f) XPS core spectra are shown in Figure 2. Au(4f) deconvoluted spectra was composed of four peaks (Figure 2c). The resolved peaks related to Au^0 ^(81.8 and 85.3 eV) exhibit the metallic nature of Au, while Au^+ ^state (83.7 and 87.4 eV) probably due to the interaction with the negatively charged graphene around Au induces a positive charge. The contribution of various spices of core level spectra is listed in Table 1.

**Table 1:** Deconvoluted contributions of various core level spectra present in Au-graphene nanocomposite

Peaks	Binding energy (eV)	Attributions	Peak area (%)
C1s (FWHM = 1.51eV)	284.6	C-C in graphene	54
	285.8	C-OH in graphene	25.4
	287.1	C-O-C in graphene	9.2
	288.6	C=O in graphene	5.7
	289.9	C(O)O in graphene	5.7
O1s (FWHM = 1.22eV)	529.3	C(O)O in graphene	14.6
	530.3	C=O in graphene	31.4
	531.1	C-O-Au of composite	27.4
	532.0	C=O in graphene	17.8
	532.9	C-OH in graphene	8.8
Au4f (FWHM= 2.03eV)	81.8, 85.3	Au4f (of Au^0^)	41.6
	83.7, 87.4	Au4f (of Au^+^)	58.4

*FWHM: Full width at half maximum

### Microstructural features induced during synthesis

**Figure 3:**
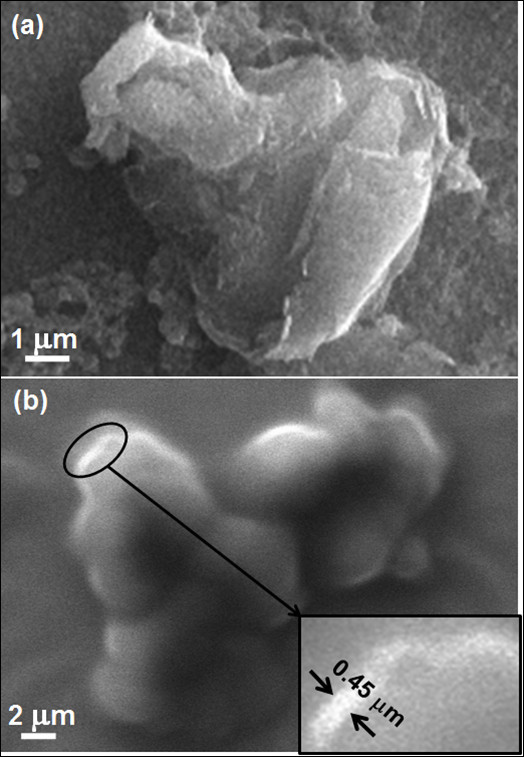
**SEM images of graphene**. Showing (**a**) an aggregate of nanosheets and (**b**) stacks of the sheets. Inset shows the edges of individual sheets.

**Figure 4:**
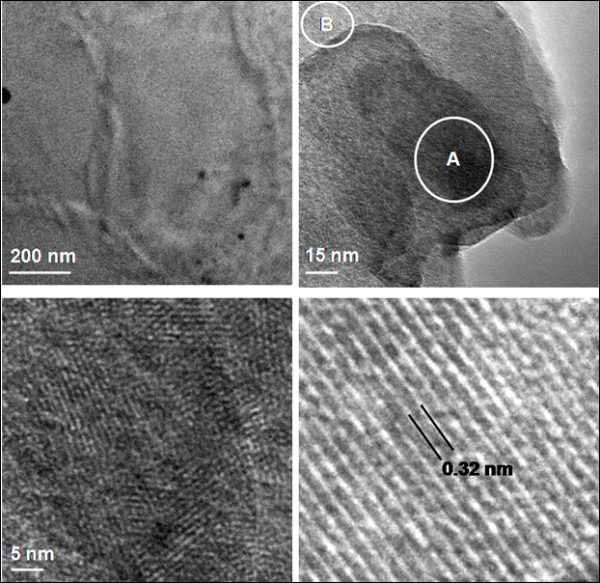
**HR-TEM micrographs of acid-functionalized grapheme**. (**a**) Sheets with wrinkle contrast, (**b**) different layers of graphene and (**c**, **d**) lattice scale fringes of graphene resolved from two different regions as marked A and B in (b).

Crumpled, folded, layers of bare graphene can be seen in the SEM image shown in Figure 3a. The SEM image of bare graphene displayed in Figure 3b shows stacks of graphene layers, bound by van der Waals forces. The thick edges of the sheets therein (inset of Figure 3b) reveal that the layers are atop each other with a thickness of about 0.45 µm. HR-TEM was employed to study the graphene and Au-graphene nanocomposites to investigate the microstructure of graphene as well as the size, shape, and distribution of Au nanoparticles in the graphene matrix (Figure 4). A conventional folded microstructure of thin graphene sheets was observed throughout the specimen (Figure 4a). The thickness of these sheets varies between 1 to 2 nm, whereas the size of these sheets is on an average between 500 nm to 1 µm (Figure 4a). A significant observation was made by resolving the graphene sheets at lattice scale. The magnified regions, marked as A and B (as indicated in Figure 4b), are displayed in Figure 4c,d, respectively. Figure 4c exhibits a cluster of graphene sheets with well-resolved fringes showing the crystalline nature of individual sheets at lattice scale, whereas Figure 4d further reveals the lattice fringe spacing of about 0.34 nm from a single sheet of a graphene. A good distribution of Au nanoparticles in the matrix phase of graphene has been delineated in the graphene-Au composite materials with a good interface between the matrix and the nanoparticle. An inset in Figure 4a exhibits the presence of carbon decorated with ultrafine dispersion of Au nanoparticle in a graphene-Au nanocomposite. Moreover, a faceted morphology of Au nanoparticle with the edges of about 30 nm clearly shows that the nanoparticle of Au is crystalline with preferred orientation (inset in Figure 4a). Since Au is characterized by a face-centered cubic crystal structure, the hexagonal-shaped particles are presumably due to the preferred growth along the 111 planes of a cubic crystal. The 111 planes of Au with graphene of *c*-axis growth of carbon lattice also justify a distinct orientation relationship and therefore a crystallographic compatibility between the carbon as matrix and the Au as second phase.

### Nano-patterning on the helium ion microscopy

**Figure 5:**
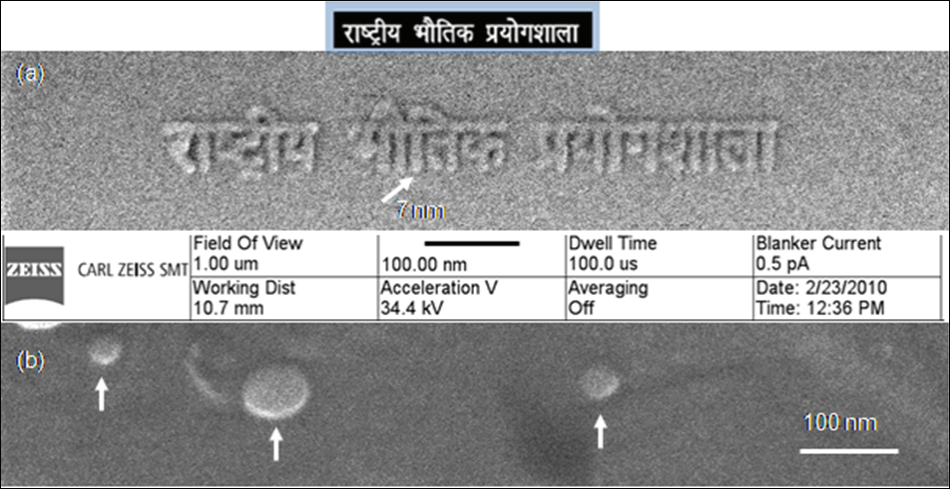
**He ion microscopy**. Showing (**a**) nano-patterning by direct write deposition and (**b**) distribution of gold particles marked with a set of arrows in graphene.

Nano-patterning by use of a high-resolution microscope is a fast developing method which facilitates *in situ *examination of the microstructure and direct write of arbitrary patterns on the given nanomaterial. HIM is showing the capability to create smaller structures than possible with other technique [15-17]. In the present work, HIM has been employed to write National Physical Laboratory in Hindi language in Devnagari script in the form of deposited carbon (Figure 5a). Combining a high-brightness gas field ion source with unique sample interaction dynamics, the He ion microscope provides images with unique contrast and complementary information to existing charged particle imaging instruments such as the SEM and TEM. Formed by a single atom at the emitter tip, the He probe can be focused to below 0.35 nm offering the highest recorded resolution for secondary electron images. The small interaction volume between the helium beam and the sample also results in images with stunning surface detail. Besides high-resolution imaging, the collimated beam of He ions can be manipulated for nano-patterning on even two-dimensional nanostructured materials like graphene. The unique combination of sub-nanometer high-resolution surface microscopy and *in situ *nano-scaled structural buildings elucidates a new field which is so far relatively unexplored in fabrication and process control of fundamentally important nano-objects like graphene. In the present work, the text ("National Physical Laboratory" in Hindi language) was created by deposition of carbon. This pattern, in the form of a bitmap, was opened up in Orion software Carl Zeiss NTS LLC, 1 Corporation Way, Peabody MA 01960, U.S.A. Subsequently, the user defined the size of the overall pattern, and the pixel size was scaled accordingly. The ion dose per pixel was also variable, being set in proportion to the gray level in the bitmap (up to 256 levels). Lettering with gaps down to 7 nm was observed. In this process, the direction of scanning was also user selectable. In another set of experiments, HIM was used to study the distribution of Au in the matrix of graphene (Figure 5b). We have noticed that spherical Au nanoparticles of size in the range of 20 to 50 nm are uniformly distributed in the matrix. A thin layer of graphene on the surface of individual Au nanoparticles is also inferred due to the presence of a glazy contrast on Au surfaces. It is important to mention that there is no deterioration at the boundaries between the matrix and the second phase.

#### Electrical properties

**Figure 6:**
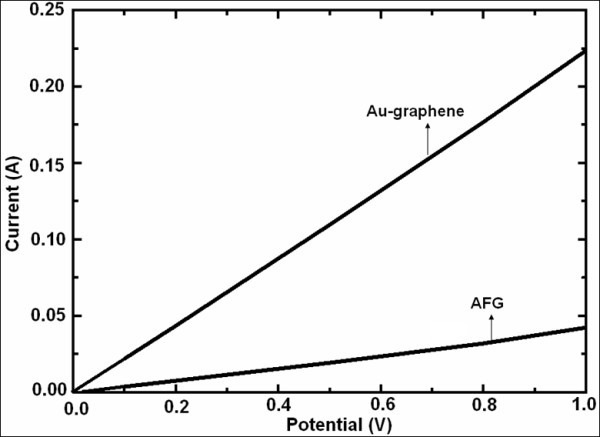
***I*-*V *characteristics of Au-graphene and blank acid-functionalized graphene**.

The *I*-*V *characteristics of Au-graphene and functionalized graphene were recorded in the following configuration: SnO_2_:F/Au-graphene/aluminum as shown in Figure 6 where respective solutions were drop casted on the SnO_2_:F-coated glass substrates. The AuNPs decoration was having a beneficial effect on the electronic conductivity of graphene. Both the films show ohmic contact with the substrate in 0 to +1 V potential region as can be seen from the linear variation of current with applied bias. For the Au-graphene composite, the conductivity was determined to be 0.49 S cm^-1 ^which is much higher than blank functionalized graphene film where the value was estimated to be 0.07 S cm^-1 ^which validates the role of interaction with AuNPs in the enhancement of conductivity. In the literature, the value of pristine graphene has been reported to be 0.2 S cm^-1 ^[9]. Here, probably due to functionalization, the value is lower. In Au-graphene, enhanced coupling occurred between AuNPs as they attached themselves onto the defect sites of graphene surface, hereby increasing the charge transfer between the two. Functional groups present on the graphene sheets served as anchors for adsorption of nanoparticles and the positively charged AuNPs as depicted earlier in XPS could easily adsorb on these negative sheets through electrostatic attraction. Moreover, even the inherent electronic conductivity of the metal NPs are higher and all these attribute to the increased conductivity in the Au-graphene nanocomposite.

## Conclusion

A simple modified Hummer's method was used to fabricate graphene and graphene-Au nanocomposites. A significant change in *I*-*V *characteristics between bare graphene and its Au incorporated nanocomposites has been noticed. An important *in situ *direct write deposition on nanosheets of graphene has been demonstrated by employing He ions inside the chamber of the microscope.

## Competing interests

The authors declare that they have no competing interests.

## Authors' contributions

SB carried out the graphene preparation and interpretation of results. MD did the lectrical measurements. AGJ carried out XPS measurements and interpretations. APS assisted in synthesis of material. AKS initiated the idea of working on present topic and subsequently compiled the data.

## References

[B1] Hermando DH Guinea F Bratas A Spin-orbit coupling in curved graphene, fullerenes, nanotubes, and nanotube caps Phys Rev B 2006 7 155426

[B2] Wallac PR The band theory of graphite Phys Rev 1947 71 622 10.1103/PhysRev.71.622

[B3] Eda G Fanchini G Chhowalla M Large-area ultrathin films of reduced graphene oxide as a transparent and flexible electronic material Nature 2008 3 270 10.1038/nnano.2008.83 18654522

[B4] Slonczewski JC Weiss PR Band structure of graphite Phys Rev 1958 109 272 10.1103/PhysRev.109.272

[B5] Vincenzo DPD Mele EJ Self-consistent effective-mass theory for intralayer screening in graphite intercalation compounds Phys Rev B 1984 29 1685 10.1103/PhysRevB.29.1685

[B6] Pasricha R Gupta S Srivastava AK A facile and novel synthesis of Ag-graphene-based nanocomposites Small 2009 5 2253 10.1002/smll.200900726 19582730

[B7] Hicks J Behnam A Ural A A computational study of tunneling-percolation electrical transport in graphene-based nanocomposites Appl Phys Lett 2009 95 213103 10.1063/1.3267079

[B8] Rafiee MA Lu W Thomas AV Zandiatashbar A Rafiee J Tour JM Koratkar NA Graphene nanoribbon composites ACS Nano 2010 4 7415 10.1021/nn102529n 21080652

[B9] Xu Y Wang Y Liang J Huang Y Ma Y Wan X Chen Y A hybrid material of graphene and poly (3,4-ethyldioxythiophene) with high conductivity, flexibility, and transparency Nano Res 2009 2 343 10.1007/s12274-009-9032-9

[B10] Xie SH Liu YY Li JY Comparison of the effective conductivity between composites reinforced by graphene nanosheets and carbon nanotubes Appl Phys Lett 2008 92 243121 10.1063/1.2949074

[B11] Wassei JK Tung VC Jonans SJ Cha K Dunn BS Tang Y Kaner RB Stenciling graphene, carbon nanotubes, and fullerenes using elastomeric lift-off membranes Adv Matter 2010 22 897 10.1002/adma.200902360 PMC4337822 20217813

[B12] Goncalves G Marques PAAP Granadeiro CM Noguiera HIS Singh MK Gracio J Surface modification of graphene nanosheets with gold nanoparticles: the role of oxygen moieties at graphene surface on gold nucleation and growth Chem Mater 2009 21 4796 10.1021/cm901052s

[B13] Muszynski R Seger B Kamat PV Decorating graphene sheets with gold nanoparticles J Phys Chem C 2008 112 526

[B14] Kim Y-K Kyung Na H Min D-H Influence of surface functionalization on the growth of gold nanostructures on graphene thin films Langmuir 2010 26 13065 10.1021/la102372z 20695544

[B15] Bell DC Contrast mechanisms and image formation in helium ion microscopy Microsc Microanal 2009 15 147 10.1017/S1431927609090138 19284896

[B16] Bell DC Lemme MC Stern LA Williams JR Marcus CM Precision cutting and patterning of graphene with helium ions Nanotechnology 2009 20 455301 10.1088/0957-4484/20/45/455301 19822934

[B17] Lemme MC Bell DC Williams JR Stern LA Baugher BWH Jarillo-Herrero P Marcus CM Etching of graphene devices with helium ion beam ACS Nano 2009 3 2674 10.1021/nn900744z 19769403

[B18] Sidorkin V Veldhoven EV Drift EVD Alkemade P Salemink H Maas D Sub-10-nm nanolithography with a scanning helium beam J Vac Sci Technol B 2009 27 L18

[B19] Winston D Cord BM Ming B Bell DC Natale WFD Stern LA Vladar AE Postek MT Mondal MK Yang JKW Berggren KK Scanning-helium-ion-beam lithography with hydrogen silsesquioxane resist J Vac Sci Technol B 2009 27 2702 10.1116/1.3250204

[B20] Alkemade P Sidorkin V Chen P Drift EVD Langen AV Maas D Veldhoven EV Scipioni L Helium ion beam processing for nano-fabrication and beam-induced chemistry Microscopy Analysis 2010 New York: Wiley 5

[B21] Zhou Y Loh KP Making patterning on graphene Adv Mater 2010 22 3615 10.1002/adma.201000436 20533420

[B22] Bell DC Lemme MC Stern LA Marcus CM Precision material modification and patterning with He ions J Vac Sci Technol B 2009 27 2755 10.1116/1.3237113

[B23] Li J Liu C-Y Ag/graphene heterostructures: synthesis, characterization and optical properties Eur J Inorg Chem 2010 1244

[B24] Henglein A Reduction of Ag(CN)2- on silver and platinum colloidal nanoparticles Langmuir 2001 17 2329 10.1021/la001081f

[B25] Brust M Walker M Bethell D Schiffrin DJ Whyman R Synthesis of thiol-derivatised gold nanoparticles in a two-phase liquid-liquid system J Chem Soc Chem Commun 1994 7 801

[B26] Lopez-Salido I Lim DC Dietsche R Bertram N Kim YD Electronic and geometric properties of Au nanoparticles on Highly Ordered Pyrolytic Graphite (HOPG) studied using X-ray Photoelectron Spectroscopy (XPS) and Scanning Tunneling Microscopy (STM) J Phys Chem B 2006 110 1128 10.1021/jp054790g 16471654

[B27] Wu XJ Zeng XC Periodic graphene nanobuds Nano Lett 2009 9 250 10.1021/nl802832m 19072128

[B28] Deng ZW Chen M Wu LM Novel method to fabricate SiO_2_/Ag composite spheres and their catalytic, surface-enhanced Raman scattering properties J Phys Chem C 2007 111 11692 10.1021/jp073632h

